# CDC25B Overexpression Stabilises Centrin 2 and Promotes the Formation of Excess Centriolar Foci

**DOI:** 10.1371/journal.pone.0067822

**Published:** 2013-07-01

**Authors:** Rose Boutros, Odile Mondesert, Corinne Lorenzo, Puji Astuti, Grant McArthur, Megan Chircop, Bernard Ducommun, Brian Gabrielli

**Affiliations:** 1 The University of Queensland Diamantina Institute, Princess Alexandra Hospital, Brisbane, Queensland, Australia; 2 Cell Cycle Unit, Children’s Medical Research Institute, Sydney, New South Wales, Australia; 3 Centre Pierre Potier, ITAV, UMS3039 CRT-CIV, Toulouse, France; 4 Faculty of Pharmacy, Universitas Gadjah Mada, Yogyakarta, Indonesia; 5 Peter MacCallum Cancer Centre, Melbourne, Victoria, Australia; 6 CNRS, LBCMCP-UMR5088, Toulouse, France; 7 CHU Purpan, Service d'Hématologie, Toulouse, France; University of Leicester, United Kingdom

## Abstract

CDK-cyclin complexes regulate centriole duplication and microtubule nucleation at specific cell cycle stages, although their exact roles in these processes remain unclear. As the activities of CDK-cyclins are themselves positively regulated by CDC25 phosphatases, we investigated the role of centrosomal CDC25B during interphase. We report that overexpression of CDC25B, as is commonly found in human cancer, results in a significant increase in centrin 2 at the centrosomes of interphase cells. Conversely, CDC25B depletion causes a loss of centrin 2 from the centrosome, which can be rescued by treatment with the proteasome inhibitor MG132. CDC25B overexpression also promotes the formation of excess centrin 2 “foci”. These foci can accumulate other centrosome proteins, including γ-tubulin and PCM-1, and can function as microtubule organising centres, indicating that these represent functional centrosomes. Formation of centrin 2 foci can be blocked by specific inhibition of CDK2 but not CDK1. CDK2-mediated phosphorylation of Monopolar spindle 1 (Mps1) at the G1/S transition is essential for the initiation of centrosome duplication, and Mps1 is reported to phosphorylate centrin 2. Overexpression of wild-type or non-degradable Mps1 exacerbated the formation of excess centrin 2 foci induced by CDC25B overexpression, while kinase-dead Mps1 has a protective effect. Together, our data suggest that CDC25B, through activation of a centrosomal pool of CDK2, stabilises the local pool of Mps1 which in turn regulates the level of centrin 2 at the centrosome. Overexpression of CDC25B may therefore contribute to tumourigenesis by perturbing the natural turnover of centrosome proteins such as Mps1 and centrin 2, thus resulting in the *de novo* assembly of extra-numerary centrosomes and potentiating chromosome instability.

## Introduction

Centrosome abnormalities occur at high rates in almost all human cancers, where a strong correlation exists between the presence of extra-numerary centrosomes, aneuploidy and chromosome instability [1–4]. The centrosome is comprised of two centrioles surrounded by an electron-dense cloud of pericentriolar material (PCM). It functions primarily to organize the microtubule network, to maintain cell polarity during interphase and to organize the bipolar spindle during mitosis for accurate chromosome segregation amongst daughter cells. For this to occur, the centrosome must duplicate itself just once during each cell division cycle. In proliferating mammalian cells, centrosome assembly usually occurs via the canonical pathway, whereby assembly of a new procentriole takes place in association with the existing mother centriole [5]. However, centrosomes can also form through the *de novo* pathway, in which random numbers of procentrioles can form inside a cloud of PCM proteins, as observed following laser ablation of centrosomes in HeLa cells [6,7]. In this pathway, exaggeration of the PCM cloud, by overexpression of pericentrin in S phase, has been shown to be sufficient to support the formation of excess daughter centrioles [8].

Centrosome duplication via the canonical pathway normally occurs in synchrony with DNA replication, and this is regulated by the activities of CDK2-cyclin E/ cyclin A complexes [9,10]. CDK-cyclin complexes in turn, are regulated by inhibitory phosphorylation of Thr14 and Tyr15 on CDK by the Wee1 and Myt1 kinases, and activatory dephosphorylation of the same sites by the CDC25 phosphatases [11]. Three CDC25 isoforms exist in mammalian cells and their overexpression, particularly CDC25A and B, are commonly reported in a wide variety of human cancers, with CDC25B overexpression in particular, being associated with more advanced disease and poor clinical outcome (reviewed in 12–14).

CDC25B localises to the centrosome throughout the cell cycle [15–18], where it participates in regulating microtubule assembly during both interphase and mitosis, and centrosome assembly during S phase [15,19]. Increased levels of CDC25B at the centrosome causes an accumulation of centrosomal γ-tubulin [15], which is essential for centriole assembly [20,21] and microtubule nucleation, in co-operation with other γ-tubulin ring complex (γTuRC) components [22]. CDC25B depletion on the other hand, results in an accumulation of G2 phase cells with significantly reduced centrosomal γ-tubulin [15] and centrin levels [23,24]. The centrosome pools of Nedd1, PCM1 and ninein are also compromised in the absence of CDC25B [24]. CDC25B may therefore be involved in centrosome targeting or in the local stability of multiple proteins involved in centriole assembly.

In the present study, we investigated the effect of CDC25B overexpression on centrin 2 and whether centrosome overduplication resulting from CDC25B overexpression may be mediated through its stabilizing effect on the centrosomal pool of centrin 2. Indeed our data indicates that centrosomal CDC25B functions to activate a local pool of CDK2 which in turn regulates the local stability of multiple centrosome proteins such as Mps1 and centrin 2, to control centrosome numbers. These findings provide insight into the pathways that drive tumourigenesis, particularly in those tumours that aberrantly overexpress CDC25B.

## Materials and Methods

### Cell Culture

U2OS and HeLa cells obtained from the American Type Culture Collection were maintained at 37°C/5% CO_2_ in Dulbecco’s modified Eagle’s Medium (DMEM) or RPMI media respectively, containing 10% fetal calf serum. U2OS cells expressing HA-CDC25B under the tetracycline-repressible promoter were cultured in the presence of 2µg/ml tetracycline. To induce HA-CDC25B expression, cells were washed 3 times in PBS and incubated in tetracycline-free media. Where indicated, cells were arrested in G1/S phase of the cycle using 4mM hydroxyurea for 36 hours prior to HA-CDC25B induction. The CDK2 inhibitor PHA533533 and the CDK1 inhibitor RO-3306 purchased from Calbiochem, were used at 1 and 9µM, respectively, as reported previously [25,26].

### Vectors and DNA transfections

GFP-tagged CDC25B WT, C487S, R506L and PACT-tagged vectors were described previously [15,24]. GFP-tagged Mps1 WT, Mps1 KD and Mps1 Δ12/Δ13 were a gift from A/Pr Fisk [27–29]. For transient transfections, U2OS cells were plated onto glass coverslips in 6-well tissue culture plates, allowed to attach overnight and then transfected with 1µg DNA using Lipofectamine 2000 reagent (Invitrogen), according to the manufacturer’s instructions. Transfected cells were fixed 24 or 48 hours later, as indicated in the text.

### RNA silencing

siRNA duplexes were transfected into HeLa cells using Lipofectamine 2000 (Invitrogen), according to the manufacturer’s instructions. Briefly, cells were seeded into 60mm culture dishes at a density of 300,000 cells per plate, and grown for 16 hours prior to transfection with 120nM siRNA for 48 hours. Control T12N scramble (Invitrogen) and CDC25B [30] siRNA duplexes were purchased from Invitrogen. MG132 was added to the culture media for the final 4 hours of the 48 hour siRNA treatment to a final concentration of 20µM.

### Immunofluorescence microscopy

For immunofluorescence studies, U2OS cells seeded onto glass coverslips and treated as indicated in the text, were fixed either in ice cold methanol for 5 minutes, or in 4% paraformaldehyde at room temperature for 20 minutes and then permeabilised for 5 minutes in 0.5% triton X-100. Cells were blocked in 5% fetal bovine serum for 1 hour at room temperature prior to antibody incubations. Anti-γ-tubulin (GTU-88) and -α-tubulin (B-5-1-2) monoclonal antibodies were purchased from Sigma Aldrich, anti-HA (Y-11) and -centrin 2 (N-17) polyclonal antibodies were from Santa Cruz Biotechnology, anti-PCM-1 was from Abcam, anti-Nek2 was from BD Transduction Laboratories and anti-centrin (20H5) was purchased from Millipore. Centrosomal CDC25B was detected using an antibody that specifically detects CDC25B phosphorylated on S230 (CDC25B-S230P) by Chk1 throughout the cell cycle, as this is currently the only reliable antibody available for the reproducible detection of endogenous CDC25B at the centrosome [24]. The rabbit polyclonal antibodies against Nedd1 and Ninein were gifts from Dr Andreas Merdes (CNRS, Pierre Fabre, Toulouse, France) and Dr Michel Bornens (Institute Curie, Paris, France), respectively. Secondary antibodies labelled with Alexa 488 or 555 were purchased from Invitrogen. DNA was visualised using 4'-6-diamino-2-phenylindole (DAPI) staining. Images were acquired using an Appotome, Laser Scanning Confocal microscope (Carl Zeiss, Germany), or a Deltavision deconvolution microscope and subsequently processed using AxioVision or Photoshop software packages.

### Western blot analyses

Cell pellets were resuspended into lysis buffer containing 50mM Tris-HCl pH7.5, 150mM sodium chloride, 1% deoxycholic acid, 1% sodium dodecyl sulfate (SDS), 1% nonidet P-40 (NP-40), 1mM DTT and phosphatase and protease inhibitors, and sonicated. Proteins were separated on 12.5% SDS-PAGE gels and transferred onto nitrocellulose membranes. Anti-HA, CDC25B, centrin 2, CDK2 and cyclin E polyclonal antibodies were from Santa Cruz Biotechnology, anti-CDK-pY15 and NPM-pT199 polyclonal antibodies were from Cell Signalling Technologies, anti-Mps1 was from Abnova and anti-α-tubulin monoclonal antibody from Sigma Aldrich. Secondary antibodies conjugated with HRP were purchased from Zymed.

### Cell cycle analysis by flow cytometry

For cell cycle analyses of U2OS-HA-CDC25B cells, cells were harvested 24 hours after treatment in the presence or absence of tetracycline and/or RO-3306 (9µM) or PHA533533 (1µM) and fixed in 70% ethanol at −20°C for 1 hour. Cells were washed once with PBS and resuspended in propidium iodide (PI) staining solution (containing 40µg/ml PI and 100µg/mL RNase A) at a density of 1 x 10^6^ cells per ml, and incubated at 37°C for 30 minutes. Cell cycle profiles were acquired with a FACSCanto flow cytometer (Becton Dickinson) using FACSDiva software (v 5.0.1) at 488 nm. Cell cycle profiles were analyzed using FlowJo software (v 7.1).

### Statistical and image analyses

All statistical analyses were performed using paired student’s *t*-tests and Microsoft Excel or Prism software packages. Image analyses of centrin fluorescence intensity were performed using the ImageJ or MetaMorph imaging software package, as described in the figure legends.

## Results

### CDC25B overexpression increases the centrosomal levels of centrin 2

**Figure 1:**
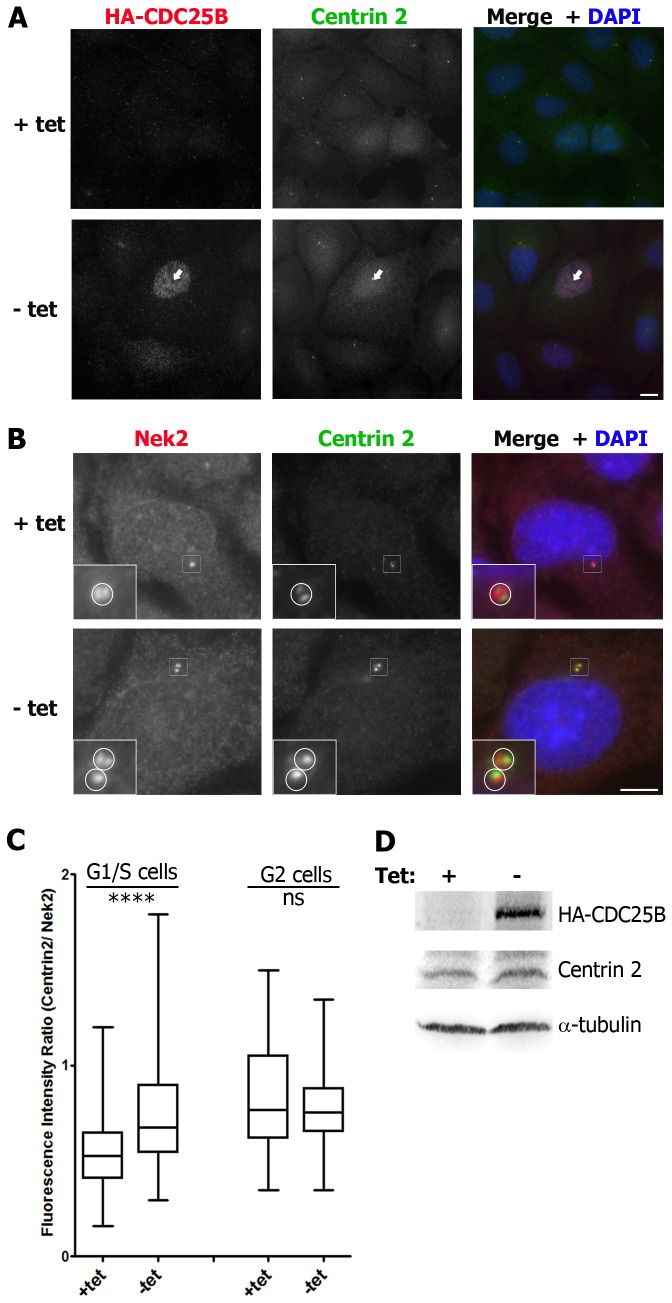
HA-CDC25B overexpression increases the level of centrin 2 at the centrosome. Asynchronous U2OS-HA-CDC25B cells were incubated in the continued presence (+tet) or absence (-tet) of tetracycline, fixed 24 hours later and co-stained for HA-CDC25B (red), centrin 2 (green) and DAPI (blue). (A) Examples of centrin fluorescence levels at the centrosome of cells expressing (-tet, arrow) or not (+tet) HA-CDC25B. Bar = 10 µm. (B, C) Analyses of centrin 2 fluorescence levels in cells expressing (-tet) or not (+tet) HA-CDC25B. (B) Cells were co-stained for centrin 2 (green) and Nek2 (red) as a marker for centrosomes. Insets are magnifications of centrosome regions (indicated by white boxes). Circles represent centrosome boundaries used to quantitate fluorescence intensities of centrin 2 and Nek2 at the centrosomes (C), using MetaMorph imaging software. (C) Box plots of the ratio of centrin 2 fluorescence intensity / Nek2 fluorescence intensity at the centrosomes of cells expressing or not HA-CDC25B. G1/S phase cells, identified as cells with a single centrosome (B, +tet) or duplicating centrosomes that were not yet separated (B, -tet), or G2 phase cells, identified as cells with 2 centrosomes that were separated, were all imaged on the same day using identical microscope and software settings. Box plots were prepared using the Prism software package and statistical analyses performed using unpaired student’s t-tests (**** P<0.0001; ns not significant, n=76-170 centrosomes). Data shown are from 1 experiment and are representative of those obtained from four independent experiments. (D) Western blot analyses of HA-CDC25B and centrin 2 levels in cells expressing or not HA-CDC25B. α-tubulin was used as a loading control.

We recently reported that CDC25B associates with centrin 2 at the centrosome and that this is important for maintaining centrosome integrity [24]. We therefore investigated the effect of CDC25B overexpression on centrosomal centrin 2 levels. In the first instance, we used a U2OS cell line (U2OS-HA-CDC25B) stably expressing HA-tagged CDC25B under the control of a tetracycline suppressible promoter [31]. Asynchronous cell populations were induced to express HA-CDC25B by removal of tetracycline from the culture media for 24 hours prior to fixation and analyses of the average fluorescence intensity of centrin 2 at the centrosome of G1/S or G2 phase cells. As shown in Figure 1 (A-B), the fluorescence intensity of centrin 2 was significantly increased at the centrosomes of G1/S phase cells expressing HA-CDC25B, compared with control cells cultured in the continued presence of tetracycline. However, there was no significant change in the centrin 2 fluorescence intensity at the centrosome of G2 phase cells (Figure 1B) or in the total centrin 2 protein pool (Figure 1C).

**Figure 2:**
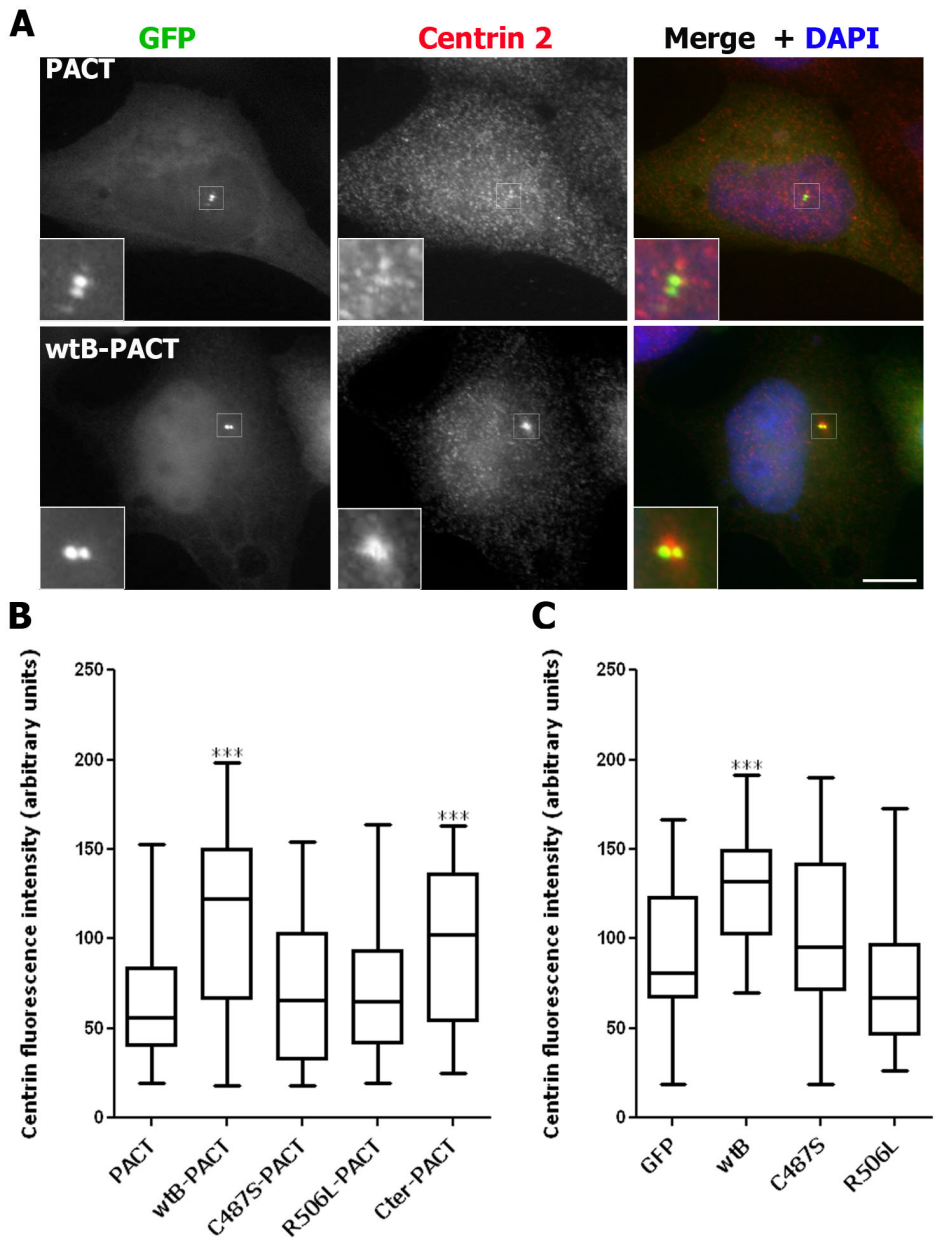
Overexpression of wild-type but not inactive CDC25B increases the level of centrin 2 at the centrosome. Asynchronous U2OS cells were transfected with pEGFP- or pEGFP-PACT-tagged wild-type (wtB), phosphatase-inactive (C487S), substrate binding deficient (R506L) or C-terminally truncated catalytically active (Cter) CDC25B vectors, fixed 24 hours later and the centrin fluorescence intensities at the centrosomes analysed as in Figure 1. (A) Examples of cells transfected with the GFP-PACT vector alone or GFP-wtB-PACT (green) and co-stained for centrin (red) and DAPI (blue). Insets are magnifications of centrosome regions (indicated by white boxes) of the transfected cell in each case. Bar = 10 µm. (B, C) Box plots of centrin fluorescence intensities at the centrosome(s) of G1/S phase cells transfected with GFP-PACT-tagged (B) or GFP-tagged (C) vectors (*** P<0.0001; n=41-59 centrosomes). Data shown are representative of those obtained from two independent experiments.

We next explored the requirement for CDC25B phosphatase activity in this centrosomal centrin 2 accumulation in the parental U2OS cell line by transiently overexpressing GFP-tagged wild-type CDC25B (wtB), the catalytically inactive (C487S) mutant [15,19], the substrate binding (R506L) mutant [32], or the catalytically active C-terminal truncation (Cter) mutant [24] (Figure 2). These CDC25B proteins were specifically targeted to the centrosome by fusion with the centrosome targeting (PACT) domain of AKAP450 [33] (Figure 2A-B), as previously shown [15]. As shown in Figure 2B, centrosomal overexpression of full-length (wtB-PACT) CDC25B significantly increased the level of centrin 2 at the centrosome, compared to cells transfected with GFP control vector. Overexpression of the C-terminal domain alone, which contains both catalytic and substrate binding regions, was sufficient to increase centrosomal centrin 2 levels (Figure 2B). In contrast, centrosomal overexpression of the catalytically inactive (C487S-PACT) or substrate binding (R506L-PACT) mutants of CDC25B had no significant effect on centrin 2 levels. Analogous results were produced in cells expressing CDC25B that harbors a mutation of a second residue within the catalytic region (Y511) (data not shown), which is also critical for CDC25B binding to its CDK-cyclin substrates [32]. Similar results were also observed with non-centrosomally targeted CDC25B (Figure 2C). These results are in agreement with our previous observations that a pool of CDC25B localises to the centrosome throughout cell division [15–18] and that catalytically active CDC25B is required for the centrosomal localization of centrin 2 [24].

### CDC25B protects centrin 2 from proteasome-mediated degradation

**Figure 3:**
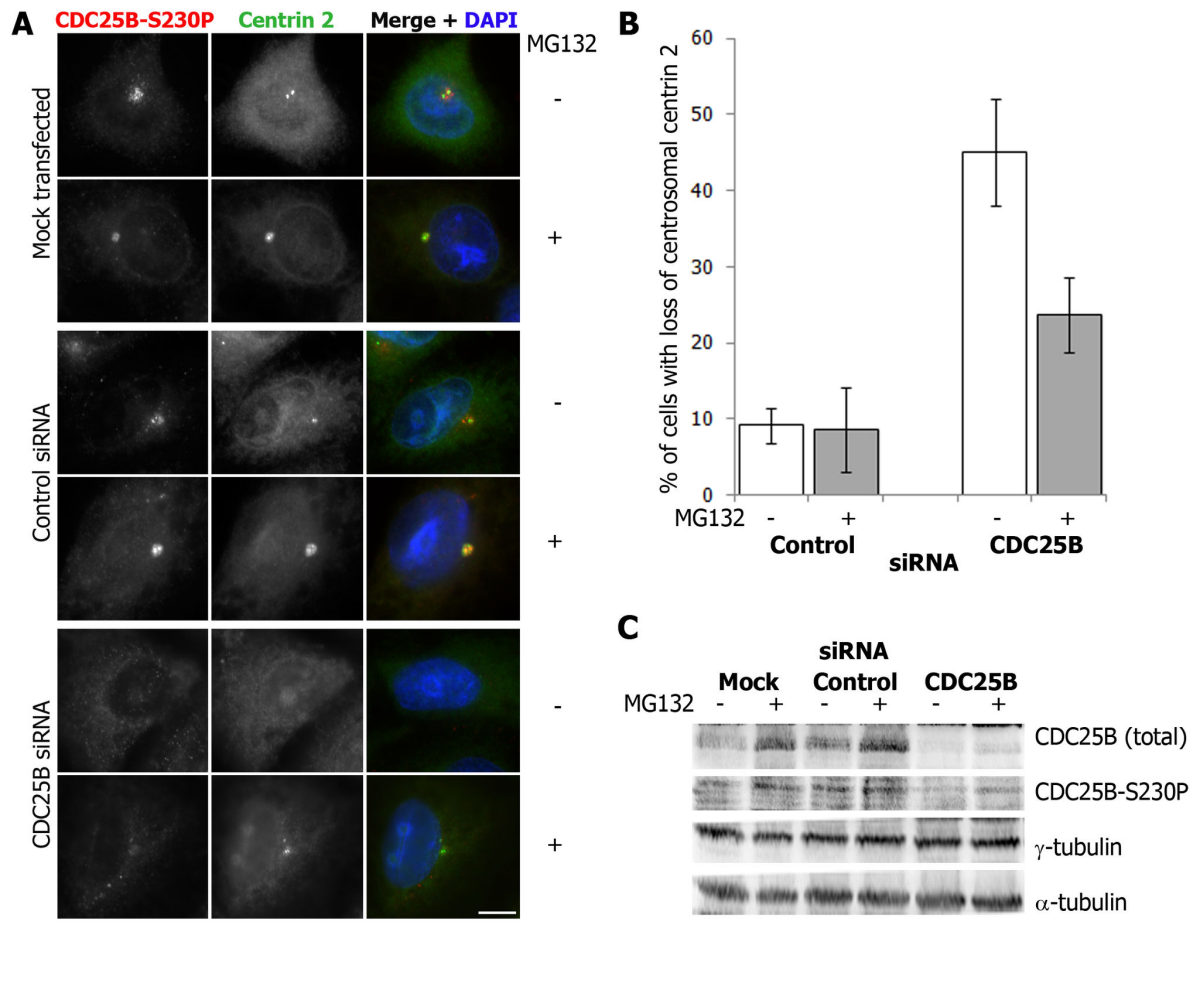
CDC25B protects centrosomal centrin from proteasome-mediated degradation. HeLa cells were either mock-transfected or treated with scrambled control or CDC25B siRNA duplexes for 44 hours followed by 4 hour treatment with 20µM MG132. (A) Examples of cells treated (+) or not (-) with MG132, and stained for CDC25B (CDC25B-S230P, red) and centrin 2 (green). Bar = 10 µm. (B) Percentage of cells treated with control or CDC25B siRNA duplexes in which centrin 2 and CDC25B-S230P (CDC25B siRNA only) were not detectable or substantially diminished at the centrosomes. Bars represent means of at least 200 cells counted from three independent experiments +/- SD. (C) Western blots of siRNA-depleted cells treated with (+) or without (-) MG132 and immunoblotted for total CDC25B, CDC25B-S230P, γ-tubulin and α-tubulin.

We have established that CDC25B plays an important role in regulating the centrosome levels of centrin 2. However, the data shown in Figures 1 and 2 do not address whether CDC25B functions in the recruitment of centrin 2 to the centrosome or in regulating centrin 2 protein stability/ turnover. We have previously demonstrated that CDC25B depletion results in a loss of centrin 2 from the centrosome in HeLa cells [24]. In order to address whether this is due to reduced centrin 2 protein stability, we sought to determine whether the loss of centrosomal centrin 2 observed in CDC25B-depleted cells [24] could be rescued by treatment with the proteasome inhibitor MG132. HeLa cells were depleted of CDC25B using siRNA for 48 hours, in the presence or absence of MG132 for the final 4 hours of siRNA treatment (Figure 3). Centrin 2 was not detectable at the centrosome of approximately 50% cells depleted of CDC25B, compared to 10% cells treated with a scrambled control siRNA (Figure 3A-B). In the presence of MG132, this loss of centrosomal centrin 2 was partially rescued (Figure 3A-B), demonstrating that CDC25B contributes to centrosomal centrin 2 stability. Western blot analyses revealed that MG132 treatment stabilized CDC25B in control cells (Figure 3B-C), in agreement with previous reports on CDC25B being regulated by proteasome mediated degradation [34]. However, MG132 treatment had no effect on the overall levels of either centrin 2 or γ-tubulin (Figure 3C), whose centrosome levels are also regulated by CDC25B [15]. Together, these data strongly suggest that CDC25B specifically protects the centrosomal pool of centrin 2 from proteasome-mediated degradation.

### CDC25B overexpression induces the formation of extra-numerary centrin 2 “foci” that accumulate other PCM components

**Figure 4:**
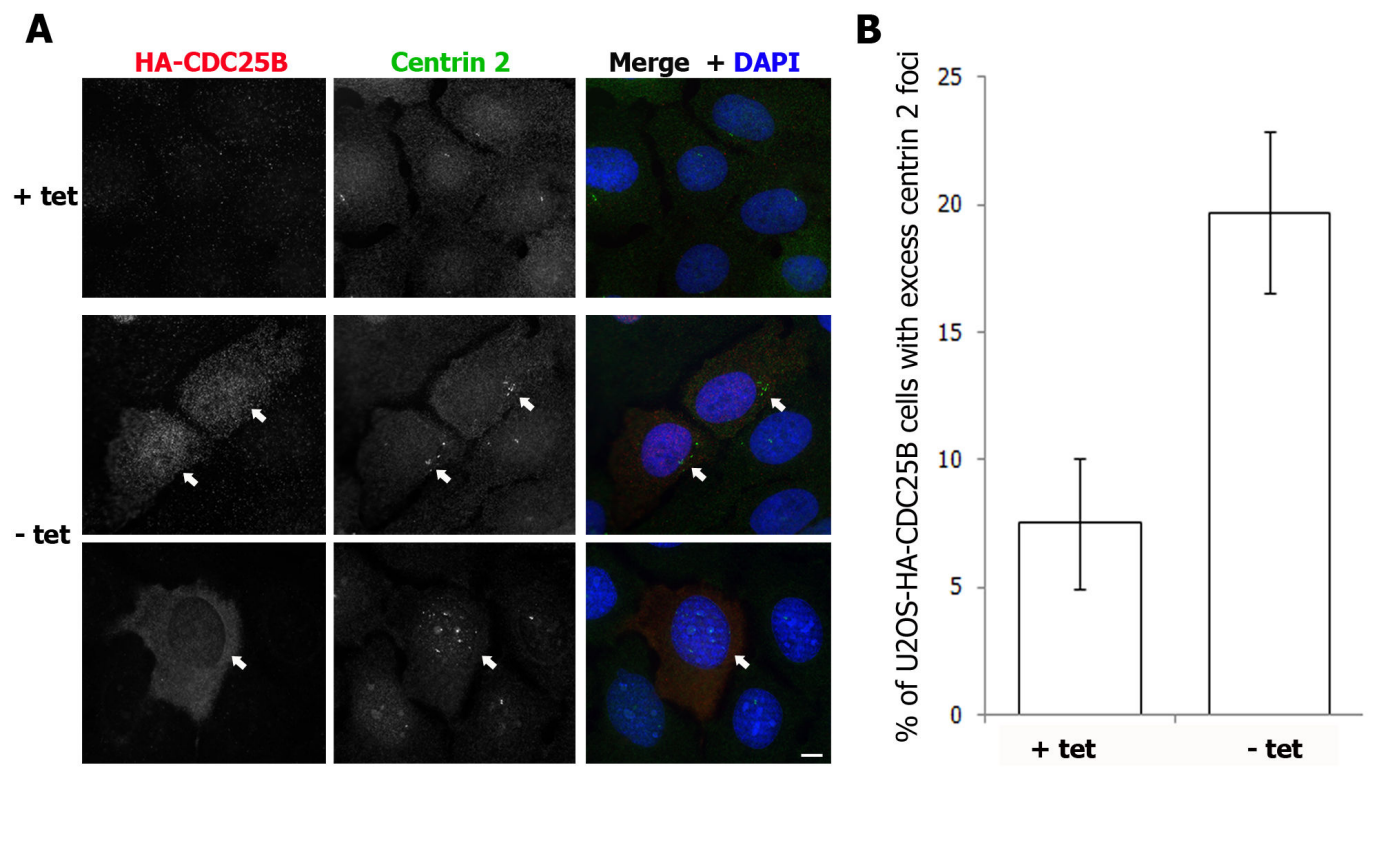
HA-CDC25B overexpression induces the formation of extra-numerary centrin 2 “foci”. Asynchronous U2OS-HA-CDC25B cells were incubated in the continued presence (+ tet) or absence (- tet) of tetracycline as in Figure 1, fixed 48 hours later and co-stained for HA-CDC25B (red), centrin 2 (green) and DAPI (blue). (A) Examples of centrin 2 foci in cells expressing (- tet, arrows) HA-CDC25B in comparison to control cells (+ tet). Bar = 10 µm. (B) Histogram plot showing the percentage of cells harbouring excess centrin 2 “foci” following 48 hours HA-CDC25B expression. Bars represent means of at least 200 cells counted from three independent experiments +/- SD.

In addition to increasing the centrosomal pool of centrin 2, we observed that overexpression of CDC25B in the U2OS-HA-CDC25B cell line promoted the formation of extra-numerary centrin 2 “foci” in approximately 20% cells expressing HA-CDC25B for 48 hours (Figure 4 and Figure S1). A time course of the appearance of these centrin foci revealed that they appeared from 2 days post-induction of HA-CDC25B expression and persisted for up to 5 days, after which time the viability of HA-CDC25B expressing cells was significantly reduced (Figure S1).

**Figure 5:**
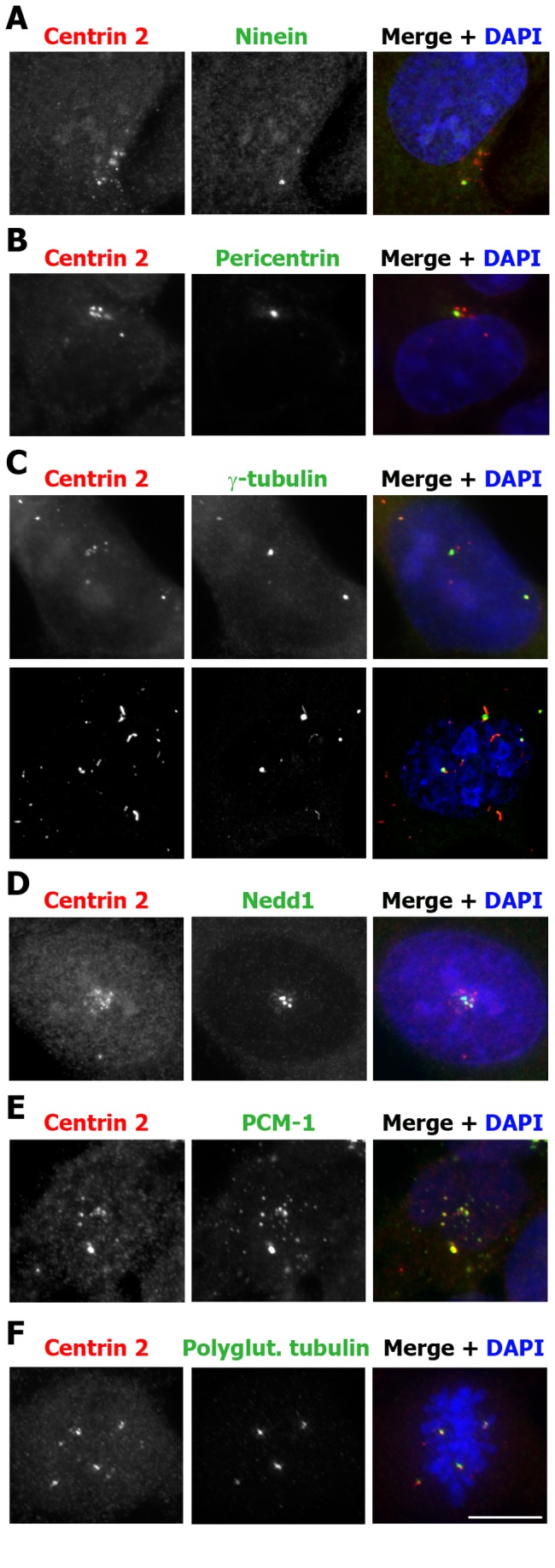
CDC25B-induced centrin 2 foci accumulate other PCM components. Asynchronous U2OS-HA-CDC25B cells were incubated in the absence of tetracycline for 48-72 hours and then fixed and co-stained for centrin 2 (red), DAPI (blue) and either Ninein (A), Pericentrin (B), γ-tubulin (C), Nedd1 (D), PCM-1 (E) or polyglutamylated (polyglut.) tubulin (F), as indicated. Images were taken using an Appotome microscope, except for C, lower panel, which is a maximum intensity projection of a z-stack taken using a laser scanning confocal microscope, to enhance the detection of γ-tubulin at distant foci. Bar = 10 µm.

The centrin 2 foci were either found to cluster around the mother centrosome or at sites that were distant from the mother centrosome within the cytoplasm, or in the nucleus (Figure 4A and Figure S2). Similar centrin “foci” or “satellites” have been observed previously, in G1/S-arrested Chinese Hamster Ovary (CHO) cells [35], in HeLa cells overexpressing a phosph-mimetic form of centrin 2 [36] and in A549 human adenocarcinoma cells following DNA damage [37]. These centrin foci were found to represent *de novo* assembled procentrioles which, in CHO cells, arise in the nucleus and are exported to the cytoplasm, where they eventually mature into centrosomes [35,37]. We therefore examined whether the excess centrin 2 foci induced by CDC25B overexpression also accumulated other components of the PCM. Consistent with CDC25B-induced centrin 2 foci representing immature centriolar satellites, ninein and Nek2, markers for the mother centrosome were never detected at these centrin 2 foci, nor was the anchoring protein pericentrin (Figure 5A-B and data not shown). However, we observed that some, but not all, of the centrin 2 foci found in cells overexpressing HA-CDC25B for 48-72 hours, accumulated the PCM components γ-tubulin, Nedd1 and PCM-1 (Figure 5C–E) and polyglutamylated tubulin, which we detect at the centrosomes in mitotic but not interphase U2OS cells (Figure 5F). Moreover, the accumulation of γ-tubulin, Nedd1 and PCM-1 occurred at centrin 2 foci that were located in the cytoplasm but not those found within the nucleus (Figure 5). Taken together, our data suggest that CDC25B overexpression induces the *de novo* formation of centriolar foci by increasing the stability of the centrosomal pools of centrin 2 (Figures 1-3), γ-tubulin [15] and potentially other centrosome proteins.

### CDC25B-induced centrin 2 foci can mature into functional centrosomes

**Figure 6:**
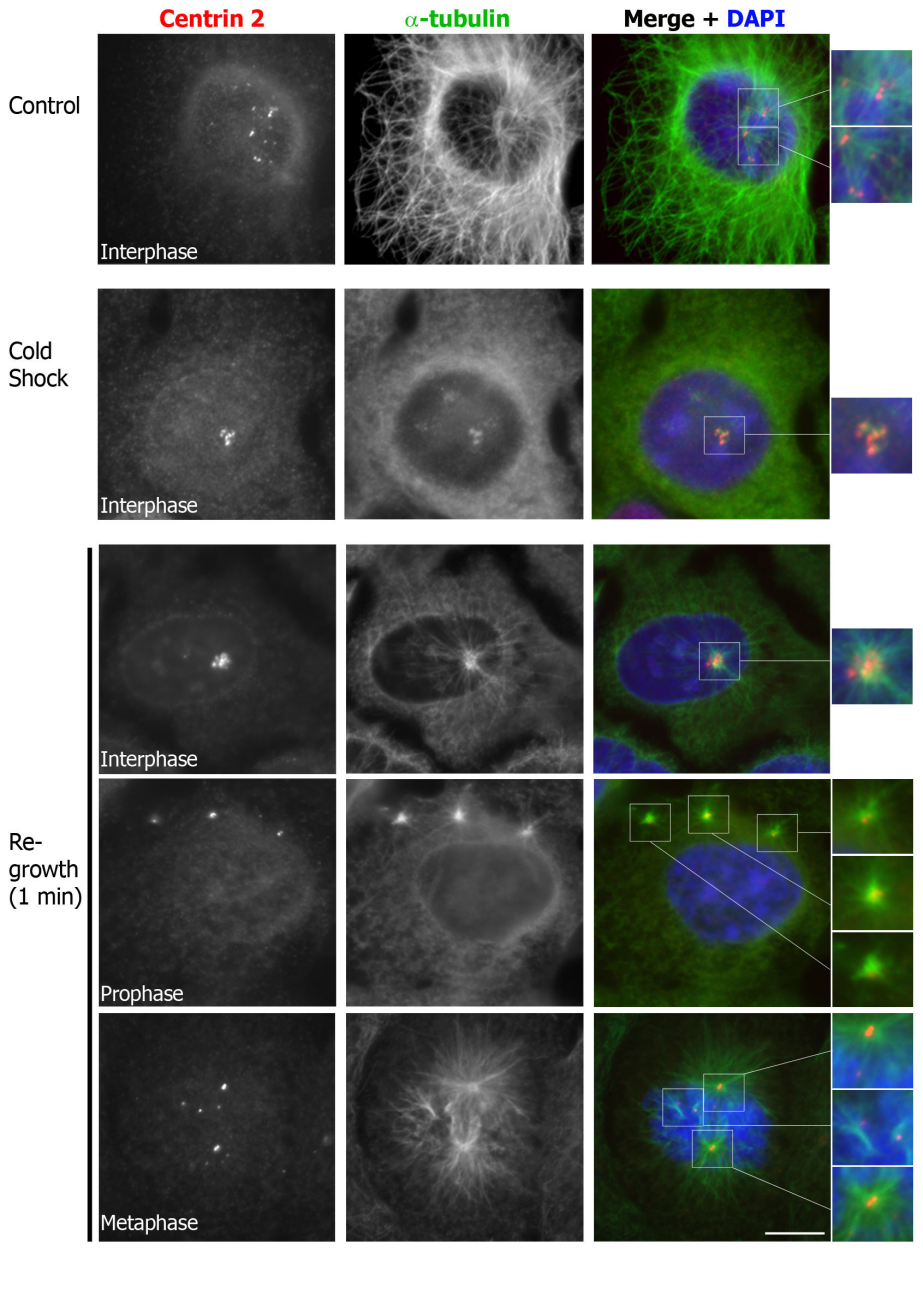
CDC25B-induced centrin 2 foci have the capacity to function as microtubule organising centres. Microtubule re-growth assay on asynchronous U2OS-HA-CDC25B cells, which were incubated in the absence of tetracycline for 5 days, and then either fixed (Control), or incubated on ice for 30 minutes, and then fixed (Cold Shock) or incubated in warm media for 1 minute (Re-growth) to allow re-assembly of microtubules. Cells were co-stained for centrin 2 (red), and α-tubulin (green), and DNA co-stained with DAPI (blue). Magnifications of the centrosomes/ centrin foci (indicated by white boxes) are shown on the right hand side. Bar = 10 µm.

To determine whether CDC25B-induced centrin 2 foci could mature into functional centrosomes, we performed microtubule re-growth assays in cells that were induced to express HA-CDC25B for up to 5 days, to allow the foci to mature. To this end, U2OS-HA-CDC25B cells were subjected to cold shock treatment from 3 to 5 days to completely deploymerise all microtubules. As shown in Figure 6, 30 minute incubation on ice was sufficient to completely disassemble the microtubule network without significant loss of centrin 2 from the primary centrosomes or the larger, more mature centriolar foci. We noted, however, that microtubule depolymerisation caused the centriolar foci to cluster around the primary centrosomes, compared to the control cells in which the foci were more dispersed (Figure 6), thus making it difficult to determine with certainty which centrioles were responsible for microtubule assembly during interphase (Figure 6, re-growth, upper panel). Nonetheless, it is possible to observe separated centrin foci in some mitotic cells, such as the examples of prophase (re-growth, middle panel) and metaphase (re-growth, lower panel) cells shown in Figure 6. In these cells, from 5 days post-HA-CDC25B induction onwards, we could observe the assembly of microtubules from the primary centrosomes in addition to some of the centriolar satellites within 1 minute following additional of warm media (Figure 6). Together, with our previous findings that CDC25B overexpression results in centrosome overduplication [15], these data suggest that overexpression of CDC25B is sufficient to promote the formation of extra centriolar foci that have the potential to recruit additional centrosome proteins and mature into functional centrosomes.

### CDC25B-induced centrin foci are dependent on the activity of CDK2 but not CDK1

**Figure 7:**
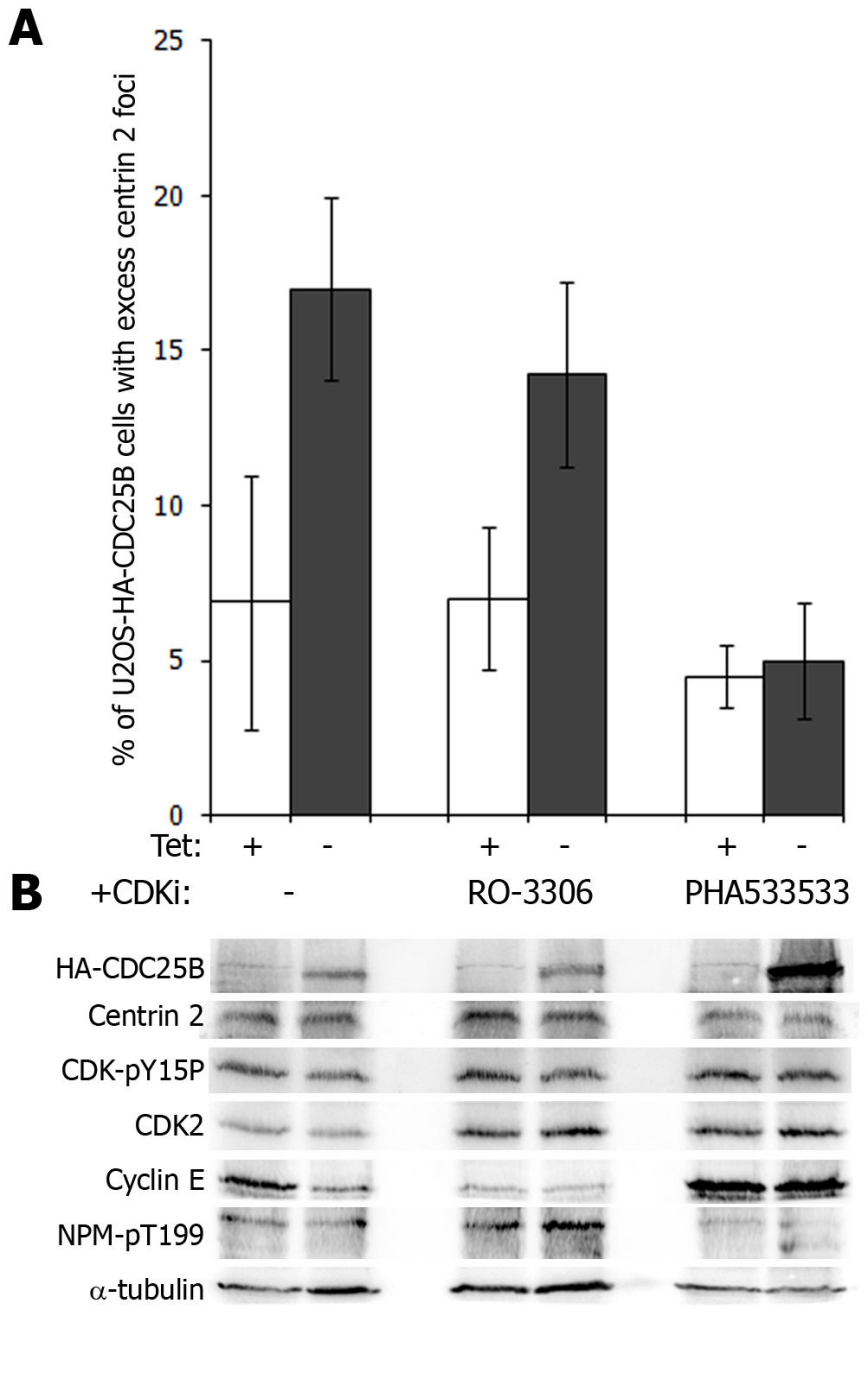
CDK2 inhibition blocks CDC25B-induced centrin 2 foci formation. (A) Asynchronous U2OS-HA-CDC25B cells were incubated in the presence (tet +) or absence (tet -) of tetracycline with either 9µM CDK1 inhibitor RO-3306 [26] or 1µM CDK2 inhibitor PHA533533 [25] for 24 hours. (A) Graph represents the percentages of cells with excess centrin 2 foci. Data shown are the means of at least 200 cells counted from three independent experiments +/- SD. (B) Western blot analyses on soluble protein extracts from cells treated as in (A). Membranes were probed for HA-tagged CDC25B, centrin 2, inactive CDK (CDK-pY15P), total CDK2-cyclin E and its substrate nucleophosmin (NPM), which is phosphorylated by CDK2-cyclin E on T199. α-tubulin was used as loading control.

CDK2 in complex with cyclin E or cyclin A has a well documented role in centrosome duplication [9,10]. As CDK-cyclins are currently the only known substrates for CDC25 phosphatases, we examined the contributions of CDK1 and CDK2 in CDC25B-mediated centriolar foci formation shown in Figures 4-6. We utilized the U2OS-HA-CDC25B system to overexpress CDC25B in the presence or absence of either the CDK1 inhibitor RO-3306 [26] or the CDK2 inhibitor PHA533533 [25], and examined the effects of these compounds on the formation of excess centrin 2 foci. Successful inhibition of CDK2 by PHA533533 was achieved, as marked by increased cyclin E and the lack of phosphorylation of the CDK2 substrate nucleophosmin (NPM) (Figure 7B). CDK2 inhibition blocked the formation of excess centrin 2 foci observed following 48 hours HA-CDC25B expression in asynchronous cells, despite the surprising increase in HA-CDC25B levels induced by this compound in the absence of tetracycline (Figure 7). This block in centrin foci formation was not due to an inhibition of S phase progression, as CDK2 inhibition in these cells caused a G2/M delay rather than G1/S arrest (Figure S3). Inhibition of CDK1 also caused a G2/M arrest (Figure S3) as expected [26], but had no effect on the number of cells with extra-numerary centrin 2 foci (Figure 7A).

Since the CDC25B-induced centriolar foci can mature into functional centrosomes and we have previously reported that CDC25B overexpression causes the formation of excess centrosomes in G1/S-arrested cells [15], we asked whether inhibition of CDK2 could also block CDC25B-induced centrosome overduplication. To address this, U2OS-HA-CDC25B cells were arrested in G1/S phase by hydroxyurea treatment and then either induced to express HA-CDC25B or not, and allowed to progress through S phase and into G2, in the presence of either CDK1 or CDK2 inhibitors. We observed that while at least 25% cells overexpressing HA-CDC25B alone or with the CDK1 inhibitor had greater than 2 centrosomes by 8 hours post-release from hydroxyurea, this percentage was reduced to background levels with the addition of the CDK2 inhibitor PHA533533 (Figure S4), indicating that CDK2 activity is required for CDC25B-induced centrosome overduplication. These data further support the hypothesis that CDC25B overexpression, through over-activation of CDK2, causes the formation of excess centriolar foci that can mature into functional centrosomes.

### CDC25B-induced centrin foci are dependent on the activity of Mps1 kinase

**Figure 8:**
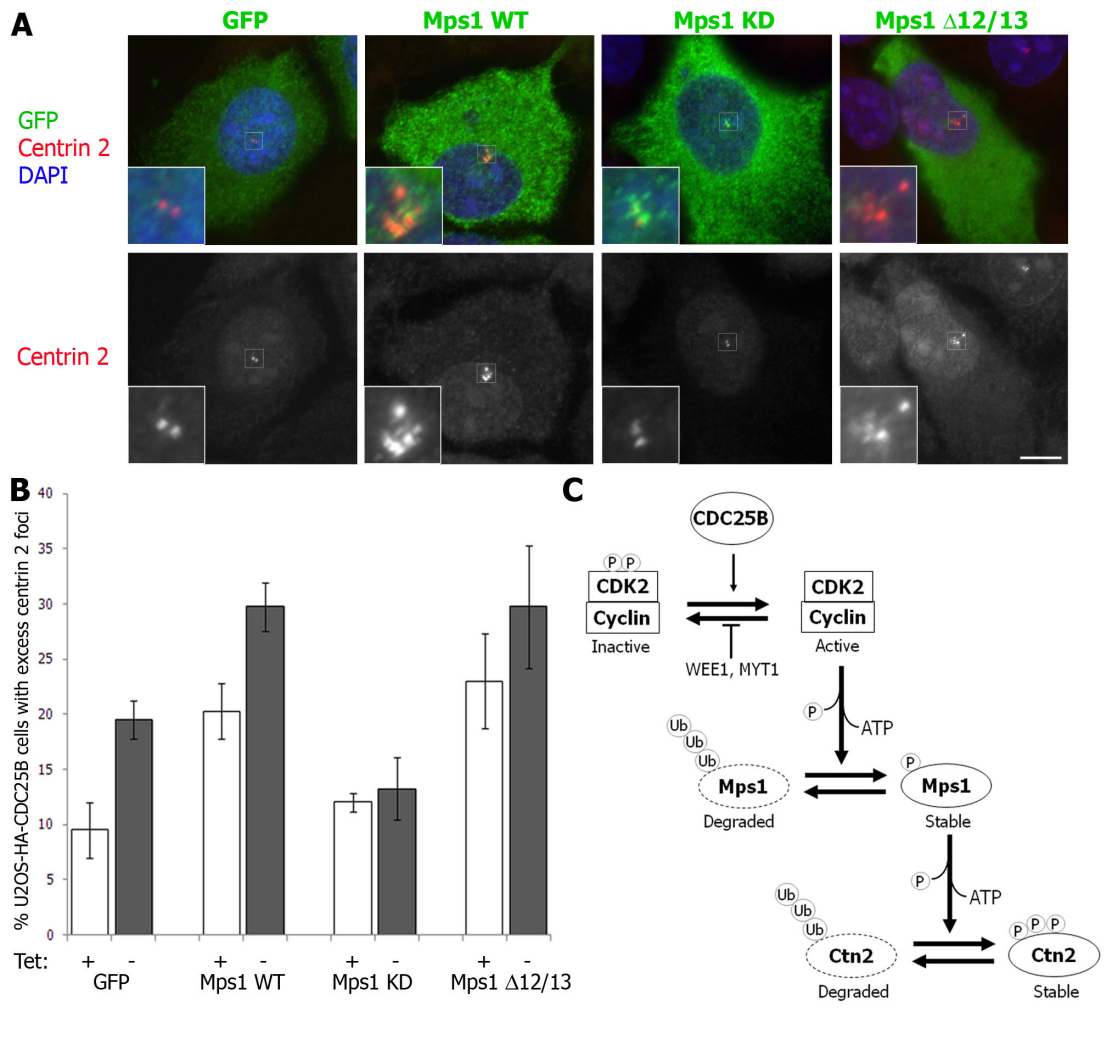
CDC25B-induced centrin foci are dependent on the activity of Mps1 kinase. Asynchronous U2OS-HA-CDC25B cells were transfected with pEGFP or pEGFP-tagged wild-type (Mps1 WT), kinase-dead (Mps1 KD) or non-degradable (Mps1 Δ12/13) forms of Mps1 and incubated in the continued presence (+ tet) or absence (- tet) of tetracycline, fixed 48 hours later and co-stained for centrin 2 (red) and DAPI (blue). (A) Examples of transfected cells cultured in the absence of tetracycline. Insets are magnifications of centrosome regions (indicated by white boxes) of the transfected cell in each case. Bar = 10 µm. (B) Histogram plot showing the percentage of cells harbouring excess centrin 2 “foci” following 48 hours transfection and HA-CDC25B expression. Bars represent means of at least 200 cells counted from four independent experiments +/- SD. (C) Model for the role of CDC25B in regulating centrin 2 protein stability through its activity towards CDK2.

The protein kinase Mps1 phosphorylates centrin 2 [36]. Overexpression of a phospho-mimetic mutant form of centrin 2 results in the accumulation of excess centrin 2 foci in HeLa cells, and this is dependent on Mps1 activity [36]. Mps1 is required for normal centrosome duplication [28]. At the start of S phase, CDK2-mediated phosphorylation of Mps1 blocks its proteasome-mediated degradation to promote centriole assembly [27]. However, unregulated inhibition of Mps1 degradation is sufficient to cause centrosome reduplication [27]. We found that, although total centrin 2 levels are not affected by CDC25B overexpression (Figure 1 and Figure S5A), Mps1 levels are increased (Figure S5A). We therefore hypothesized that Mps1 may contribute to CDC25B-induced effects on centrin 2 centrosomal protein levels and centriolar foci formation. To test this, we transiently overexpressed wild-type Mps1 (Mps1 WT) in addition to non-degradable (Mps1 Δ12/13) [29] and kinase-dead (Mps1 KD) [28] forms of Mps1 in U2OS-HA-CDC25B cells that were induced to express HA-CDC25B for 48 hours post Mps1 transfection and examined their effect on centrin 2 foci formation. We found that overexpression of both WT and Δ12/13 non-degradable Mps1 significantly increased the percentage of cells with excess centrin 2 foci after 48 hours HA-CDC25B overexpression, compared to GFP-transfected cells (Figure 8A-B). This increase was not entirely dependent on CDC25B overexpression, since a similar increase was observed in the absence of HA-CDC25B overexpression (Figure 8B). However, expression of the KD form of Mps1 blocked the formation of centrin 2 foci in HA-CDC25B overexpressing cells to background levels (Figure 8B). The total levels of centrin 2 were not affected by Mps1 overexpression, indicating that the formation of excess centrin 2 foci are not due to an overall increase in centrin 2 protein levels (Figure S5B). Thus, while CDC25B is dependent on the activity of Mps1 for the induction of centrin 2 foci, Mps1 can induce centrin 2 foci independently of CDC25B overexpression, as reported previously [36]. However, it should be noted that U2OS-HA-CDC25B cells also express endogenous CDC25B, which is likely to be sufficient to maintain normal CDK2 activity at the centrosome in uninduced (+tet) cells and is therefore likely contributing to the stability of Mps1 WT protein. These data are therefore consistent with the hypothesis that CDC25B-induced centrin 2 protein stability and centriolar foci formation occurs indirectly, through an effect on CDK2 and Mps1 kinases (Figure 8C).

## Discussion

Centrosome abnormalities are a hallmark of human cancer cells [1–4]. Overexpression of CDC25 phosphatases also occurs at a high rate in a wide range of cancers, and the CDC25B isoform in particular, is associated with more aggressive disease [13]. We have previously found that CDC25B overexpression in the human osteosarcoma cell line U2OS, causes centrosome amplification [15] and that CDC25B associates with centrin 2 [24], an integral component of both mature and assembling centrioles [38]. In the present study, we report that CDC25B overexpression significantly increases the concentration of centrin 2 at the centrosome, without significantly affecting the total protein level. We also found that CDC25B overexpression induces the formation of extra-numerary centrin 2 foci. Similar foci or procentrioles have been reported previously to arise from ectopic expression of GFP-tagged centrin 1 or centrin 2. The GFP-centrin 1 foci were found to originate in the nucleus before being exported to the cytoplasm where they accumulated additional PCM components over time, and matured into centrosomes [7,35]. Consistent with this, was our observation that excess centrin 2 foci that formed as a result of CDC25B overexpression, were localized either within the nucleus or in the perinuclear region of the cytoplasm. Furthermore, some of these centrin 2 foci, when localized in the cytoplasm, accumulate the PCM and centriollar satellite protein PCM-1 [39] as well as the γTuRC components, γ-tubulin and Nedd1 [22,40]. However, ninein, which exclusively marks the mature/ mother centrosome [41] and pericentrin were not detected on centrin foci for up to 72 hours after expression of CDC25B. This is consistent with our previous findings that while CDC25B depletion by siRNA causes a loss of centrosomal γ-tubulin and Nedd1 and dispersal of PCM-1, no effect is observed on either ninein or pericentrin [24], suggesting that CDC25B regulates the stability/ protein levels of a only a subset of PCM components. The accumulation of this protein subset at CDC25B-induced centrin 2 foci, like those induced by ectopic expression of GFP-centrin 1 in hydroxyurea-arrsted CHO cells [35] or centrin 2 in cells treated with various DNA damaging agents [37], suggests that at least some of these foci represent immature centrosome precursors, or procentrioles. Whether or not these can mature into functional microtubule organizing centres is probably largely dependent on the local availability of their associated regulatory components, such as Mps1 in the case of centrin 2.

The centrin 2 stabilizing effect of CDC25B overexpression is dependent on CDC25B catalytic activity and substrate binding capacity. We therefore investigated the contribution of CDK activity and found that specific inhibition of CDK2 from the time of CDC25B overexpression, could block both the formation of excess centrin 2 foci (this study) and centrosome overduplication that occurs following release from G1/S arrest in CDC25B overexpressing cells [15]. These results are also in agreement with those of Prosser et al. [35] who found that treatment of HU-arrested CHO cells overexpressing GFP-Centrin 1 with CDK2 inhibitors, could prevent the formation of excess centrin 2 foci and the duplication of γ-tubulin positive centrosome structures. We further demonstrated an essential role for kinase active Mps1, which phosphorylates centrin 2 to protect it from proteasome-mediated degradation [36], in the formation of CDC25B-induced centrin 2 foci.

We therefore propose that CDC25B and centrin 2 are targeted to the centrosome together, and once at the centrosome, centrin 2 levels are regulated by a balance of proteasome-mediated degradation and a CDC25B-CDK2-Mps1-mediated pathway for protecting centrin 2 from proteasome degradation. In this way, overexpression or increased stability of centrin 2 is sufficient to promote the *de novo* formation of procentriole structures that have the capacity to mature into functional centrosomes.

The CDC25B-CDK2-substrate nexus that we have investigated at the centrosome is therefore likely to regulate the local concentrations of multiple centrosome proteins, including centrin 2 and γ-tubulin and, in so doing could potentially function as a rate limiting factor in centrosome assembly. Overexpression of CDC25B and/or CDK2-cyclin E/A, which are commonly found in a wide range of human cancers, may therefore be sufficient, through centrosomal substrates like Mps1, to promote the formation of extra-numerary centriolar foci that can mature into functional centrosomes and potentiate chromosome misegregation and aneuploidy, through the formation of aberrant multipolar mitotic spindles.

## Supporting Information

Figure S1 Centrin 2 foci accumulate from 2 days post-expression of HA-CDC25B. Bar graph showing the accumulation of centrin 2 foci in cells expressing HA-CDC25B, from 1 to 5 days after the removal of tetracycline. Bars represent means +/- SD from 2 independent experiments.
(TIF)

Figure S2 Centrin 2 foci in the nucleus of U2OS-HA-CDC25B cells. An example of a U2OS-HA-CDC25B cell in which HA-CDC25B expression has been induced for 48 hours prior to fixation and co-staining for HA-CDC25B, Centrin 2 and DAPI. Images represent a z-stack taken for each channel using a Deltavision deconvolution microscope and a 100X oil objective. Bar = 10 µm.
(TIF)

Figure S3 CDK1 and CDK2 inhibitors cause G2/M but not G1/S arrest. DNA histogram profiles of U2OS-HA-CDC25B cells cultured in the presence (+tet) or absence (-tet) of tetracycline for 24 hours in the presence of either the CDK1 inhibitor RO-3306 or the CDK2 inhibitor PHA533533.
(TIF)

Figure S4 CDK2 inhibition blocks CDC25B-induced centrosome overduplication. U2OS-HA-CDC25B cells were arrested in G1/S phase with 36h hydroxyurea treatment and released for up to 8hrs either in the presence of tetracycline (+ tet) or in the absence of tetracycline (- tet) alone or with CDK1 (- tet + RO-3306) or CDK2 (- tet + PHA533533) inhibitors. Cells were co-stained for HA and γ-tubulin and the percentages of cells with more than 2 centrosomes scored. Bars represent means of at least 200 cells counted from three or more independent experiments +/- SD.
(TIF)

Figure S5 Increased Mps1 levels following HA-CDC25B induction. Asynchronous U2OS-HA-CDC25B cells were incubated for 48 hours in the continued presence (+ tet) or absence (- tet) of tetracycline (A) and following transfection with GFP and GFP-tagged Mps1 variants (B). Western blot analyses are of HA-CDC25B, Mps1 or GFP-Mps1 and centrin 2 levels. α-tubulin was used as a loading control.
(TIF)
