# Supplementary figures and images for: CDC25B Overexpression Stabilises Centrin 2 and Promotes the Formation of Excess Centriolar Foci
Source: PLoS One. 2013 Jul 1;8(7):e67822. doi: 10.1371/journal.pone.0067822 (PMC3698172; doi:10.1371/journal.pone.0067822)

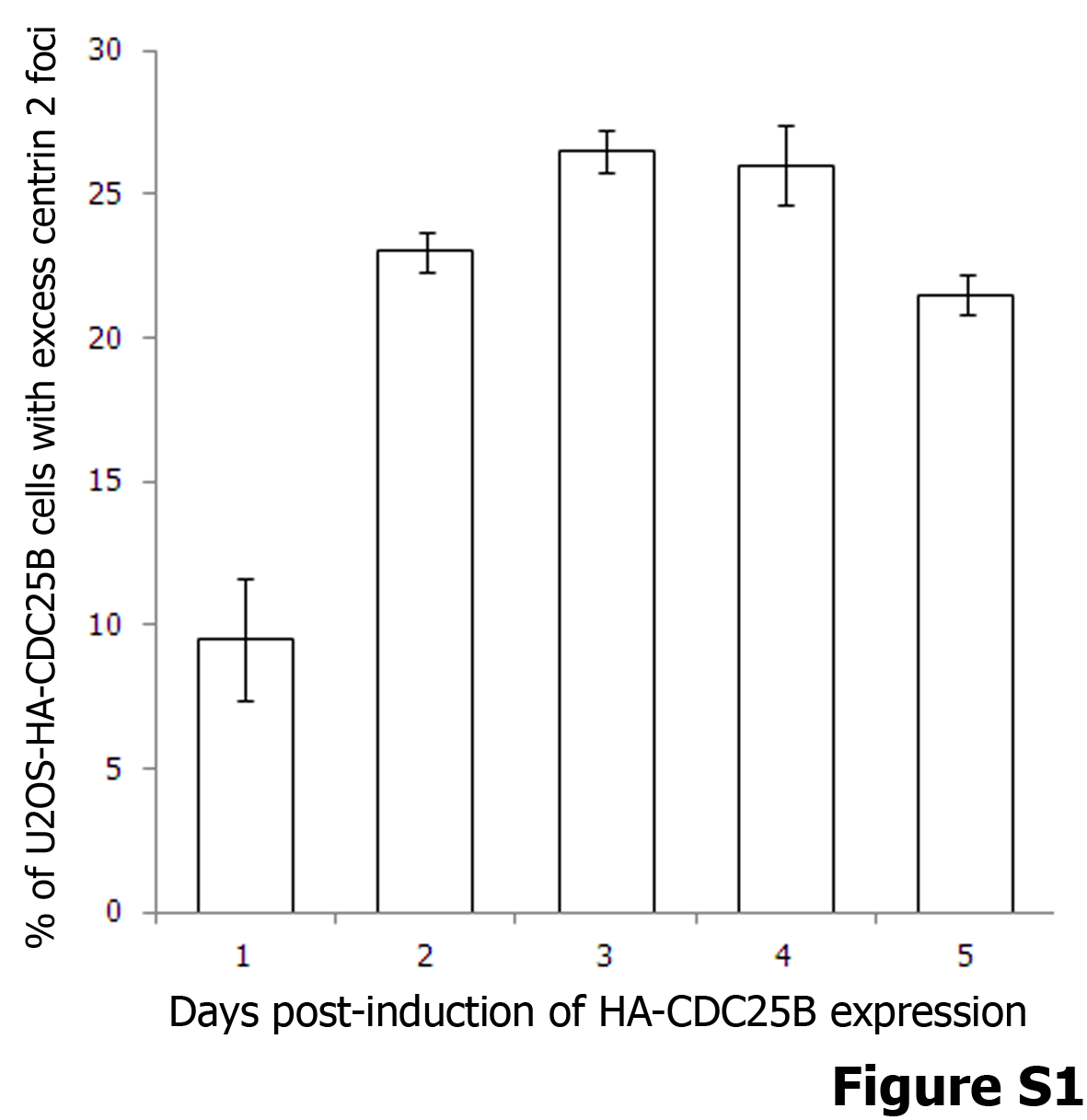

Supplement: Figure S1 — Bar graph showing the accumulation of centrin 2 foci in cells expressing HA-CDC25B, from 1 to 5 days after the removal of tetracycline. Bars represent means +/- SD from 2 independent experiments. (TIF) [file pone.0067822.s001.tif]

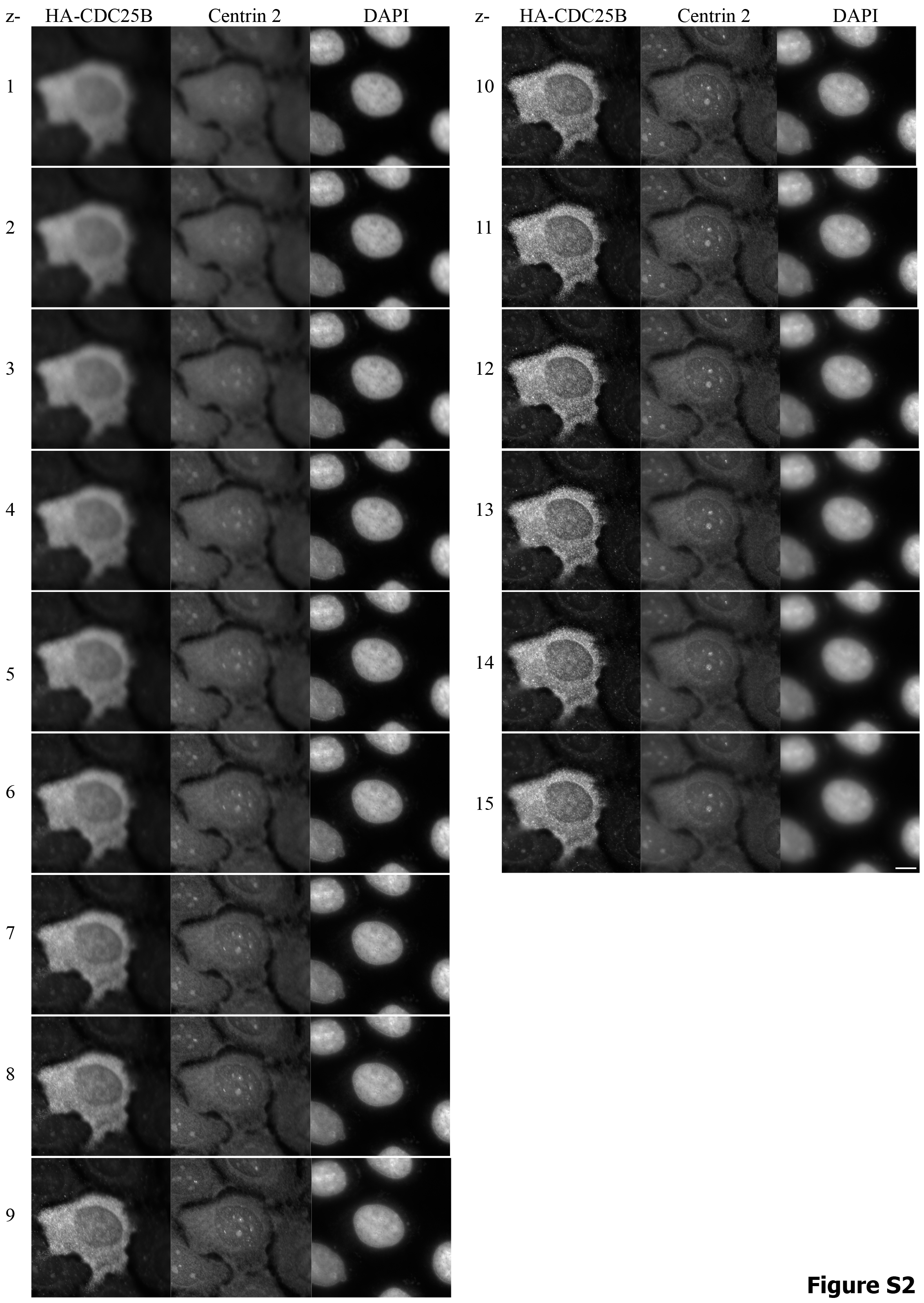

Supplement: Figure S2 — An example of a U2OS-HA-CDC25B cell in which HA-CDC25B expression has been induced for 48 hours prior to fixation and co-staining for HA-CDC25B, Centrin 2 and DAPI. Images represent a z-stack taken for each channel using a Deltavision deconvolution microscope and a 100X oil objective. Bar = 10 µm. (TIF) [file pone.0067822.s002.tif]

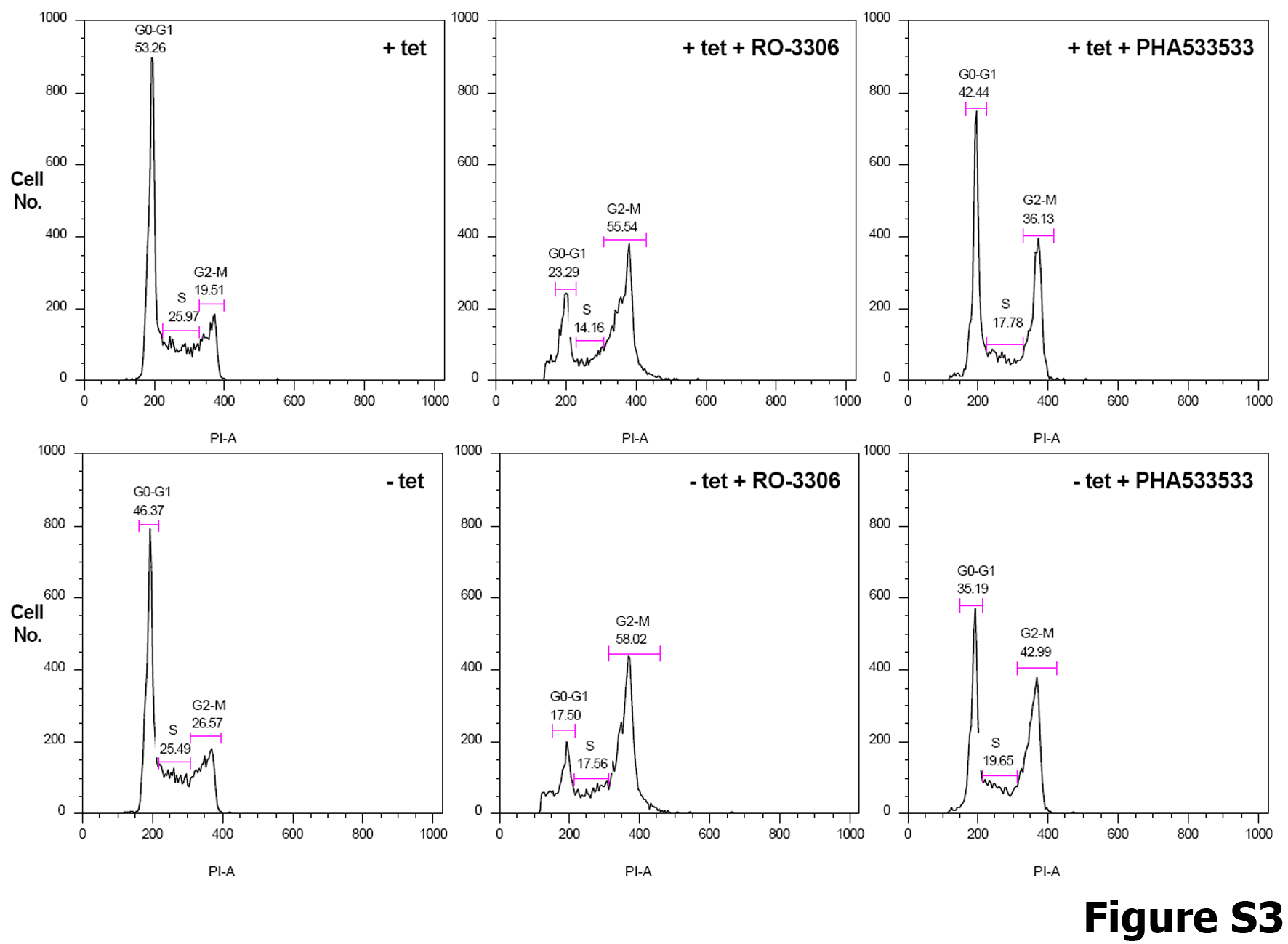

Supplement: Figure S3 — DNA histogram profiles of U2OS-HA-CDC25B cells cultured in the presence (+tet) or absence (-tet) of tetracycline for 24 hours in the presence of either the CDK1 inhibitor RO-3306 or the CDK2 inhibitor PHA533533. (TIF) [file pone.0067822.s003.tif]

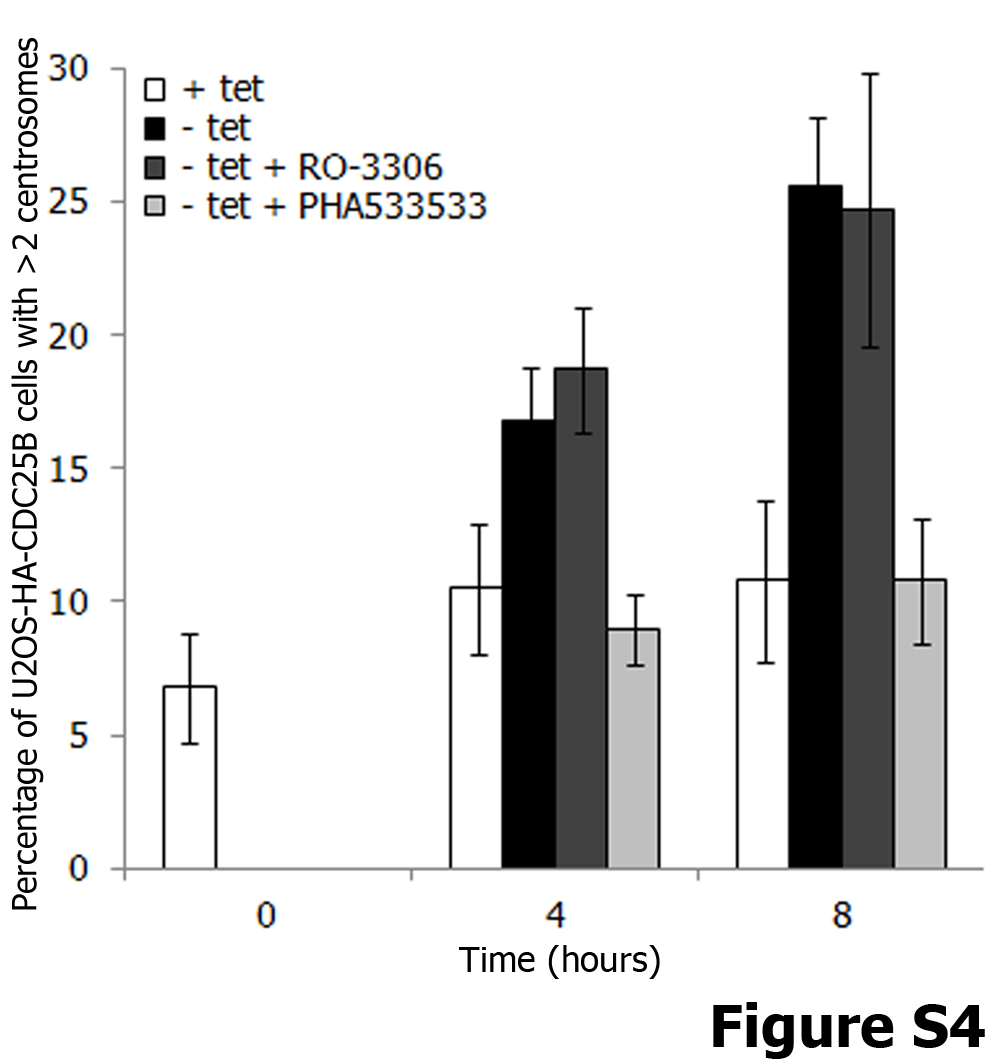

Supplement: Figure S4 — U2OS-HA-CDC25B cells were arrested in G1/S phase with 36h hydroxyurea treatment and released for up to 8hrs either in the presence of tetracycline (+ tet) or in the absence of tetracycline (- tet) alone or with CDK1 (- tet + RO-3306) or CDK2 (- tet + PHA533533) inhibitors. Cells were co-stained for HA and γ-tubulin and the percentages of cells with more than 2 centrosomes scored. Bars represent means of at least 200 cells counted from three or more independent experiments +/- SD. (TIF) [file pone.0067822.s004.tif]

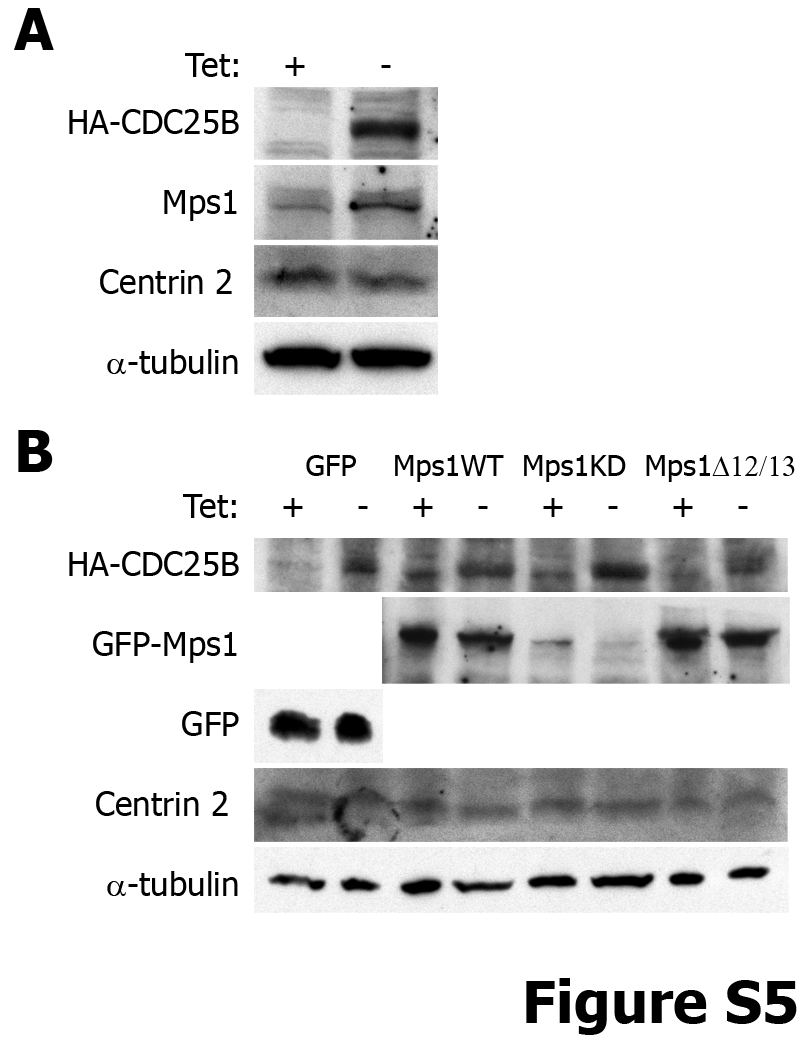

Supplement: Figure S5 — Asynchronous U2OS-HA-CDC25B cells were incubated for 48 hours in the continued presence (+ tet) or absence (- tet) of tetracycline (A) and following transfection with GFP and GFP-tagged Mps1 variants (B). Western blot analyses are of HA-CDC25B, Mps1 or GFP-Mps1 and centrin 2 levels. α-tubulin was used as a loading control. (TIF) [file pone.0067822.s005.tif]
